# Assessment and Diagnosis of Cognitive Disorders: Toward Integrated and Multidimensional Diagnostic Frameworks

**DOI:** 10.3390/diagnostics16060897

**Published:** 2026-03-18

**Authors:** Elena Cecilia Rosca

**Affiliations:** 1 Department of Neurology, Victor Babes University of Medicine and Pharmacy Timisoara, Eftimie Murgu Sq. No. 2, 300041 Timisoara, Romania; rosca.elena@umft.ro or roscacecilia@yahoo.com; Tel.: +40-746-173-794; 2 Department of Neurology, Clinical Emergency County Hospital Timisoara, Bd. Liviu Rebreanu, No. 152, 300736 Timisoara, Romania

## 1. Introduction

Cognitive disorders represent one of the most significant and rapidly evolving challenges in contemporary medicine. Affecting individuals across the lifespan and arising from neurological, systemic, developmental, and psychiatric conditions, cognitive impairment increasingly influences functional independence, quality of life, and long-term health outcomes. As global populations age and survival improves among patients with chronic diseases, clinicians face growing diagnostic complexity, with cognitive symptoms frequently emerging at the intersection of multiple biological and environmental influences.

Historically, the assessment of cognitive disorders has been grounded in careful clinical observation. Classical neurological practice emphasizes that cognitive syndromes reflect patterns of network dysfunction rather than specific pathological entities alone, underscoring the central role of structured history-taking, informant reports, and domain-oriented cognitive examination [1]. This clinical framework remains fundamental, as the purpose of cognitive assessment is not merely to quantify impairment but to localize dysfunction, guide diagnostic reasoning, and determine the need for further investigation.

At the same time, contemporary research has refined the concept of mild cognitive impairment (MCI) as an intermediate stage between normal cognitive aging and dementia, representing a critical window for risk stratification and early intervention [2]. Despite widespread recognition of MCI, considerable heterogeneity persists in diagnostic approaches, reflecting differences in assessment tools, biomarker availability, and clinical interpretation. Analyses of international guidelines emphasize that neuropsychological evaluation combined with biomarker assessment constitutes the most consistently recommended strategy, although standardization remains incomplete [3].

This perspective introduces the Special Issue “Assessment and Diagnosis of Cognitive Disorders”, which brings together contributions reflecting this ongoing transformation in cognitive diagnostics. The articles in this Special Issue collectively illustrate how the field is moving beyond traditional symptom-based paradigms toward multidimensional diagnostic frameworks that integrate systemic physiology, molecular biology, cognitive phenotyping, and computational innovation.

## 2. Expanding Diagnostic Dimensions: From Systemic Disease to Cognitive Phenotypes

A major insight emerging from contemporary research is that cognitive dysfunction rarely exists in isolation from broader physiological processes. Several contributions in this Special Issue demonstrate how systemic disease directly influences cognitive outcomes, challenging the historical separation between neurological and general medical disorders.

Vastag et al. [4] show that patients with fibrosing interstitial lung diseases exhibit measurable cognitive impairment that is associated with pulmonary functional decline and disease severity. This finding supports the concept of a lung–brain axis in which chronic hypoxemia, inflammation, and metabolic stress contribute to neural vulnerability. Similarly, Staicu et al. [5] demonstrate that postoperative delirium and cognitive dysfunction following cardiac surgery arise through complex interactions among inflammatory activation, perioperative physiological stress, and patient-specific risk factors. Together, these studies reinforce the view that neuroinflammation and systemic stress represent central mechanisms linking bodily illness to cognitive decline.

These observations align with broader epidemiological evidence emphasizing vascular and systemic contributors to cognitive impairment across aging populations [2]. Cognitive disorders, therefore, increasingly require interdisciplinary evaluation integrating neurological assessment with perspectives from internal medicine, cardiology, pulmonology, and critical care.

Diagnostic complexity is further illustrated by studies examining clinically overlapping syndromes. Aoun Sebaiti et al. [6] identify distinct cognitive phenotypes differentiating myalgic encephalomyelitis/chronic fatigue syndrome from multiple sclerosis despite shared symptoms, underscoring the importance of detailed neuropsychological profiling. Using large population-based data, Yen et al. [7] demonstrate an increased likelihood of dementia diagnosis among patients with epilepsy while also revealing how healthcare utilization and clinical surveillance influence detection rates. These findings highlight that diagnosis reflects not only underlying biological processes but also patterns of clinical observation and healthcare access.

Expanding beyond adult populations, further research [8] demonstrates that visuospatial organizational strategies are closely associated with grammatical abilities in children with specific language impairment. Their results support network-based models of cognition in which language, executive function, and perceptual organization emerge from interacting neural systems rather than isolated domains. Collectively, these studies illustrate a shift from categorical disease models toward cognitive phenotyping grounded in multidimensional, lifespan-oriented perspectives, underscoring the need for diagnostic frameworks that integrate systemic, developmental, and neurocognitive dimensions.

## 3. Biomarkers, Genomics, and Computational Approaches in Cognitive Diagnosis

In parallel with advances in clinical characterization, the contributions included in this Special Issue highlight transformative progress in biological and technological diagnostic tools. Biomarkers hold considerable promise for improving diagnostic precision; however, successful translation into clinical practice requires methodological rigor, validation, and standardization. Urbano et al. [9] address this challenge by comparing neurofilament light chain measurements across analytical platforms, demonstrating strong correlations alongside systematic differences that necessitate harmonization models. Their findings underscore that diagnostic reliability depends not only on biomarker validity but also on measurement consistency across laboratories and clinical settings.

These challenges reflect broader guideline observations indicating that biomarker integration is increasingly essential but remains incompletely standardized within current diagnostic pathways [3]. Future diagnostic frameworks will likely rely on multimodal biomarker strategies combining molecular, imaging, physiological, and behavioral indicators rather than single measures, thereby enabling a more comprehensive characterization of cognitive disorders.

Genomic approaches further expand diagnostic possibilities. Research demonstrates the clinical impact of whole-exome sequencing in a large cohort of patients with neurodevelopmental delay, identifying numerous pathogenic and previously undescribed variants [10]. Such findings exemplify the transition toward genotype-informed classification, in which molecular mechanisms refine disease boundaries and support precision diagnostics beginning in early life. By revealing shared genetic substrates across clinically heterogeneous presentations, genomic analyses contribute to reconceptualizing cognitive disorders as biologically interconnected spectra rather than discrete entities.

Technological innovation represents an additional transformative dimension. The systematic review by Halkiopoulos et al. [11] illustrates how deep learning applied to neuroimaging enables automated detection of complex neural patterns associated with emotional and cognitive processing. Increasingly, artificial intelligence systems are capable of integrating heterogeneous clinical information, mirroring real-world diagnostic reasoning. Recent large-scale evidence has demonstrated the feasibility of such approaches: an artificial intelligence model that was trained on multimodal datasets, including demographics, medical history, neuropsychological testing, functional assessments, and neuroimaging, achieved high diagnostic accuracy in differentiating cognitive states and multiple dementia etiologies. Additionally, the model improved clinician performance when used as a decision-support tool [12]. Notably, AI-assisted evaluations showed substantially higher diagnostic accuracy compared with clinician-only assessments, highlighting the potential of computational systems to augment, rather than replace, expert clinical judgment [12].

Importantly, these technological advances should not be interpreted as substitutes for clinical expertise. Instead, they extend the interpretative capacity of clinicians by synthesizing complex multidimensional data that exceed traditional analytic limits. Consequently, the convergence of biomarkers, genomics, and artificial intelligence does not entail a departure from clinical neurology; rather, it is a progression toward a data-informed and integrated model of cognitive diagnosis. In this model, biological signals and clinical reasoning work in tandem to facilitate the earlier and more precise identification of cognitive disorders.

## 4. Toward Integrated Cognitive Diagnostics: Future Directions

Taken together, the studies included in this Special Issue outline a coherent vision for the future of cognitive disorder diagnosis. Cognitive impairment increasingly emerges as a multidimensional phenomenon shaped by dynamic interactions among neural networks, systemic physiology, genetic architecture, behavior, and environmental influences. Within this evolving framework, the clinical cognitive examination remains indispensable, providing the interpretative foundation through which biomarkers and technological tools acquire clinical meaning and diagnostic relevance [1].

Early identification of cognitive decline, particularly at transitional stages such as mild cognitive impairment, represents a critical opportunity for intervention, risk modification, and patient counseling [2]. Rather than relying on isolated diagnostic markers, future models of care will likely integrate longitudinal monitoring, multimodal biomarkers, neuropsychological profiling, and personalized risk prediction to support preventive and therapeutic decision-making across the disease continuum.

Equally important is ensuring the accessibility and applicability of emerging diagnostic approaches across diverse healthcare systems. Advances in genomics and artificial intelligence must be accompanied by ethical oversight, clinician education, data transparency, and equitable implementation strategies to maximize clinical benefit while minimizing disparities in access to innovation. As diagnostic technologies become increasingly data-driven, maintaining the central role of clinical reasoning and patient-centered evaluation will remain essential.

Recent developments in multimodal artificial intelligence systems capable of integrating clinical, cognitive, and imaging data further illustrate the direction of this transformation, demonstrating how computational tools may enhance diagnostic accuracy and support clinician decision-making within real-world practice [12]. Such approaches exemplify the emergence of collaborative diagnostic models in which human expertise and machine learning operate synergistically rather than competitively.

The present Special Issue, therefore, reflects a broader transition within neuroscience and clinical medicine, from descriptive classification toward precision cognitive neurology. By integrating clinical observation with biological, genomic, and computational innovation, the field moves closer to achieving earlier detection, improved diagnostic accuracy, and individualized management strategies for individuals experiencing cognitive disorders. Ultimately, the future of cognitive diagnostics will depend not on any single technological advance, but on the successful integration of multidisciplinary knowledge into coherent, patient-centered diagnostic frameworks.

## Data Availability

No new data were created or analyzed in this study. Data sharing is not applicable to this article.

## Abbreviations

The following abbreviations are used in this manuscript:

MCI	Mild cognitive impairment
